# Comparative transcriptome analysis provides insights into dwarfism in cherry tomato (*Solanum lycopersicum* var. *cerasiforme*)

**DOI:** 10.1371/journal.pone.0208770

**Published:** 2018-12-07

**Authors:** Md Abdur Rahim, Hee-Jeong Jung, Khandker Shazia Afrin, Ji-Hee Lee, Ill-Sup Nou

**Affiliations:** 1 Department of Horticulture, Sunchon National University, Suncheon, Republic of Korea; 2 Department of Genetics and Plant Breeding, Sher-e-Bangla Agricultural University, Dhaka, Bangladesh; 3 Center for Horticulture Seed Development of Golden Seed Project, Sunchon National University, Suncheon, Republic of Korea; Key Laboratory of Horticultural Plant Biology (MOE), CHINA

## Abstract

Tomato, which can be eaten as a vegetable or fruit, is one of the most popular and nutritionally important crops around the world. Although most plants of the cherry tomato cultivar ‘Minichal’ have a normal phenotype, some plants have a stunted phenotype with reduced plant height, leaf size, and fruit size, as well as altered leaf and fruit shape. To investigate the molecular mechanisms underlying these differences, we generated RNA-seq libraries from pooled leaf samples of 10 normal (N) and 10 stunted (S) plants. Using the Illumina sequencing platform, we obtained a total of 115.45 million high-quality clean reads assembled into 35,216 genes and 35,216 transcripts. A total of 661 genes were differentially expressed between N and S plants. Of these, 420 differentially expressed genes (DEGs) were up-regulated, and 221 DEGs were down-regulated. The RNA-seq data were validated using quantitative reverse-transcription PCR. Enrichment analysis of DEGs using the Kyoto Encyclopedia of Genes and Genomes (KEGG) showed that the enriched pathways were involved in steroid biosynthesis, homologous recombination, and mismatch repair. Among these, three genes related to steroid biosynthesis, including *3BETAHSD/D2*, *DIM* and *DWF5* were down-regulated in S compared to N. Of these, *DIM* and *DWF5* are known to be involved in brassinosteroid biosynthesis. Our results thus provide a useful insight into dwarfism in cherry tomato, and offer a platform for evaluating related species.

## Introduction

Cultivated tomato (*Solanum lycopersicum* L.) is nutritionally rich, economically important, and widely grown around the world. It is ranked as the second most-consumed vegetable after the potato [1]. Tomato can be consumed fresh or in processed food items such as ketchup, paste, juice, pizza sauce, and soup. The ripe tomato fruit is abundant in lycopene, a red carotenoid pigment that has antioxidant properties, which help to protect against heart diseases, and lung and prostate cancer [2–5]. It also contains other carotenoids, including beta-carotene, neurosporene, lutein, and zeaxanthin, which support the human immune system [6]. Tomato is also a good source of vitamins, minerals and bioactive phenolic compounds, including vitamin C, vitamin K, tocopherols, folate, and potassium [7].

Cherry tomato (*Solanum lycopersicum* var. *cerasiforme)* is an ancestor of the domesticated form of cultivated tomato [8]. The content of bioactive compounds is generally higher in cherry-type tomatoes than in large ones [9], and fresh cherry tomatoes contain higher levels of nutrient and phenolic compounds than their processed products [9]. Therefore, the rate of consumption of fresh tomato fruit is increasing rapidly, and cherry tomatoes, in particular, are becoming increasingly popular as a fresh salad food because of their high nutritional quality.

In this study, we characterized a stunted phenotype of the cherry tomato cultivar ‘Minichal’, which exhibits defective growth and development, including reduced internode length, a highly branched inflorescence and reduced fruit size compared to the normal ‘Minichal’ phenotype. This dwarfism ultimately reduces the economic value of this tomato cultivar.

Several previous reports have demonstrated that mutations in hormone biosynthesis and signaling genes can result in dwarfism in plants. For example, Koornneef and van der Veen [10] characterized *GA5* (*GA20ox1*) mutants in *Arabidopsis*. *GA5* is involved in gibberellic acid (GA) biosynthesis, and its mutation leads to plant dwarfism. Timpte et al. [11] described a mutation in *axr2*, which affects an auxin responsive protein and causes dwarfism in *Arabidopsis* characterized by reduced cell length and number in both hypocotyls and inflorescences, and also by reduced epidermal cell size. Notably, exogenous treatment with auxin was able to rescue the mutant phenotype [12].

In addition, several brassinolide (BL) steroids, which are collectively known as brassinosteroids (BRs) [13,14] are crucial for normal growth and development in plants [15]. BL is the most active form of BR, and is the end product of the BR biosynthesis pathway [13,16]. BRs are involved in a variety of physiological processes, including promotion of cell elongation, cell differentiation, retardation of senescence, promotion of ethylene biosynthesis, modulation of stress responses, and regulation of gene expression [17]. They are biosynthesized through two alternate pathways; the early and late C-6 oxidation pathways [15,17,18], have been studied in plant species including *Arabidopsis*, pea, rice, and tomato [18]. The enzymes catalyzing the BR biosynthesis pathway have been particularly well characterized in *Arabidopsis*, as have BR biosynthesis mutants that result in a dwarf phenotype, including *det2* [19], *dwf1* [20,21], *cpd* [22], *dwf4* [23,24], *dwf5* [25], *dwf7* [26], and *sax1* [27], and BR signaling and perception mutants [18]. Two BR biosynthesis dwarf mutants, *BR‐deficient dwarf1* (*brd1*) and *ebisu dwarf* (*d2*), which exhibit stem and leaf elongation abnormalities, have been reported in rice [28]. In addition, a dwarf mutant with reduced BR levels, *lk*, has been reported in pea, and exogenous application of brassinolide restores it to a normal growth phenotype [18]. In cultivated tomato, two dwarf mutants, *dumpy* (*dpy*) and *dwarf* (*d*), have been reported, both of which are defective in BR biosynthesis [12].

Until now, little was known about the molecular mechanisms underlying dwarfism in cherry tomato. To gain insight into these molecular mechanisms, we used the Illumina sequencing platform to carry out transcriptomic analysis of leaves from normal (N) and stunted (S) tomato plants of the cultivar ‘Minichal’. We identified differentially expressed genes that might be involved in dwarfism of this cherry tomato cultivar ‘Minichal’. We further validated their expression pattern by qRT-PCR. These results provide a basis for identifying the key genes involved in tomato dwarfism.

## Materials and methods

### Plant materials

**Fig 1:**
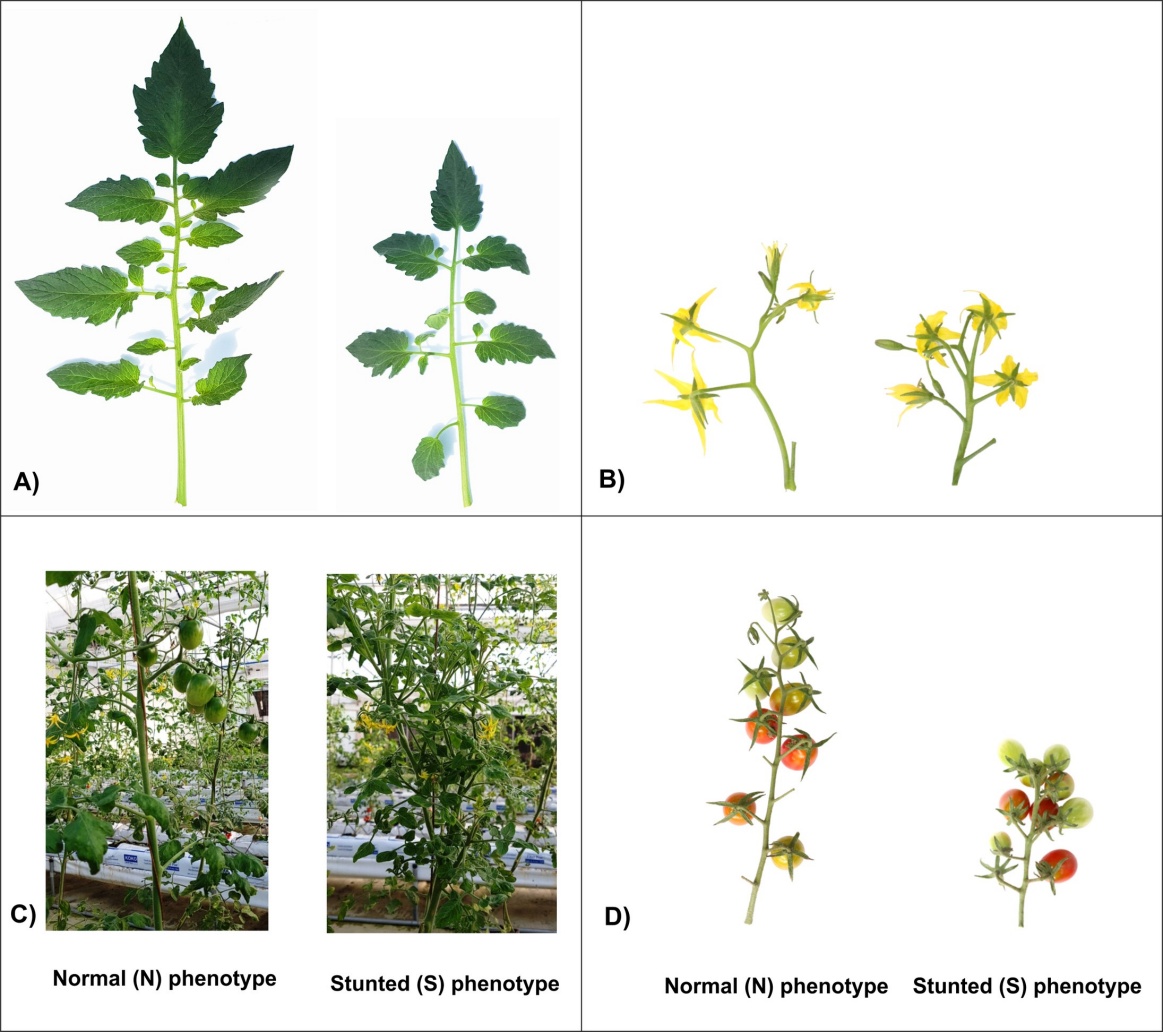
Phenotypes of normal (N) and stunted (S) plants of the cherry tomato cv. ‘Minichal’. A) Leaves; B) inflorescences; C) fruits; D) mature plants.

Two different phenotypes of the tomato cultivar ‘Minichal’, which included normal (N) plants with regular growth and development, and stunted (S) plants with reduced plant growth and development, were used (Fig 1). These lines were grown in a glasshouse at the Department of Horticulture, Sunchon National University, Suncheon, Republic of Korea. Young leaves were sampled from 10 individual plants for each phenotypic category (N and S). Leaves were pooled and frozen in liquid nitrogen before storing them at –80°C until required.

### RNA extraction and library construction for transcriptome analysis

Total RNA was isolated from 100 mg finely powdered leaf tissues using the RNeasy Mini Kit (Qiagen, USA). The quantity and integrity were checked with a NanoDrop spectrophotometer (NanoDrop Technologies, Wilmington, Delaware, USA) and an Agilent 2100 BioAnalyzer (Agilent Technologies, Palo Alto, CA, USA). Two RNA-seq libraries were constructed by Theragen Bio Institute (Suwon, South Korea) using the TruSeq RNA Library Prep Kit (Illumina Inc.) and RNA samples with a RIN (RNA integrity number) greater than 7. RNA sequencing was performed using an Illumina HiSeq 2000 platform (Illumina Inc.). RNA sequencing data were analyzed according to the method described by Trapnell et al. [29].

### Quantification of expression patterns and differentially expressed genes

Reference genome and gene model annotation files for tomato (*Solanum lycopersicum*) were retrieved from the Ensembl database (https://plants.ensembl.org/). Clean reads were mapped to the reference genome using TopHat (v.2.1.1; http://ccb.jhu.edu/). Assembled genes were searched against the Swiss-Prot database and Gene Ontology (GO) categories. Gene expression patterns and differential expression were determined using Cufflinks (v.2.0.1; http://cufflinks.cbcb.umd.edu/), as previously reported by Trapnell et al. [29]. The expression level was normalized by the number of fragments per kilobase of exon per million mapped reads (FPKM). Differentially expressed genes (DEGs) were detected using DEGseq [30] with an adjusted *p* < 0.005 and *q* < 0.05. All DEGs were subjected to GO analysis and Kyoto Encyclopedia of Genes and Genomes (KEGG) pathway enrichment analysis using WebGestalt [31] and DAVID (https://david.ncifcrf.gov/).

### Validation of RNA-seq data by qRT-PCR

The expression patterns of eight genes were selected for further validation by quantitative reverse transcription PCR (qRT-PCR). cDNA was synthesized from 2 μg of high-quality total RNA using SuperScript III (Invitrogen, Gaithersburg, MD). The qRT-PCR reaction was carried out using 50 ng cDNA and a gene-specific primer (S1 Table) with 2x SyGreen Mix Lo-ROX (qPCRBIO; PCR Biosystems, London, UK) and a LightCycler 96 instrument (Roche, Mannheim, Germany). The reaction conditions were: 95°C for 5 min, then 50 cycles at 95°C for 10 s, 60°C for 10 s, and 72°C for 15 s. Cq values obtained from qRT-PCR were analyzed with LightCycler 96 software (Roche, Germany). The mean normalized expression was determined by the comparative 2^−ΔΔCt^ method [32], where *Elongation factor-1alpha* (*EF-1alpha*) was used as an internal control gene for *Solanum lycopersicum*.

## Results

### Overview of RNA sequencing

**Fig 2:**
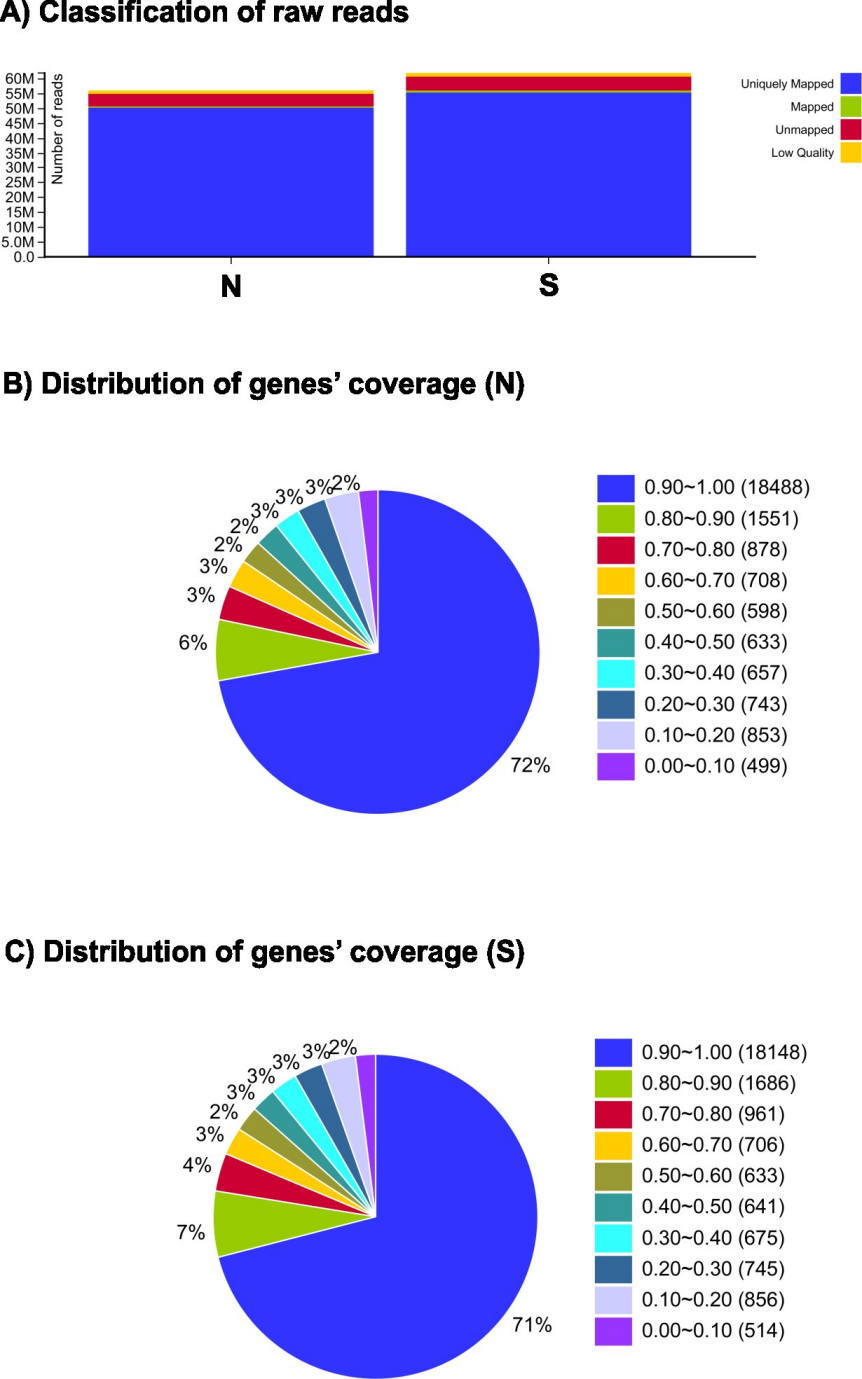
Quality raw reads and gene coverage of normal (N) and stunted (S) phenotypes of the cherry tomato cv. ‘Minichal’ using RNA-seq. A) Classification of raw reads; B) distribution of genes’ coverage for normal pool (N); C) distribution of genes’ coverage for stunted pool (S).

Leaves at similar stages of growth were collected and pooled from normal (N) and stunted (S) tomato plants for RNA isolation. Their transcriptomes were profiled using the Illumina sequencing platform. We obtained 117.995 million paired-end raw reads (Table 1). Subsequently, adapters, low-quality reads, and ambiguous reads were removed (Fig 2A). A final total of 115.450 million high-quality clean reads were obtained (54.755 and 60.695 million for N and S, respectively) (Table 1).

**Table 1:** Overview of transcriptome sequencing and assembly to the tomato (*Solanum lycopersicum*) reference genome.

Samples	Raw reads	Clean reads n (%)	Total mapped n (%)	Uniquely mapped n (%)	READ 1/ READ 2	Strand(+)/ strand(-)	Splice reads n (%)	Q20 (%)	GC (%)
**Normal (N)**	56,088,460	54,754,874 (97.6)	50,766,351 (92.7)	50,139,657 (91.6)	25,334,107/ 24,805,550	24,955,309/ 25,184,348	16,552,209 (30.2)	96.60	43.47
**Stunted (S)**	61,907,020	60,695,416 (98.0)	56,095,234 (92.4)	55,446,956 (91.5)	28,004,215/ 27,442,741	27,602,386/ 27,844,570	18,465,751 (30.4)	96.81	43.55
**Total**	**117,995,480**	**115,450,290**							
**Total number**	**Transcripts**	**Genes**							
	35,216	35,216							

A total of 97.6% reads from N plants, and 98.0% reads from S plants, were mapped to the *S*. *lycopersicum* reference genome (Ensembl). On average, 91.6% and 91.5% reads, respectively, uniquely mapped to the reference database. High-quality clean reads were assembled into 35,216 transcripts and 35,216 genes (Table 1). Among the annotated genes, 72% and 71% had 90–100% coverage in N and S tomato libraries, respectively (Fig 2B and 2C), indicating that the distributions of reads were similar between tomato libraries. Both the samples had Q20 scores (indicating Phred-like quality) greater than 96%, indicating the high quality of the RNA sequencing. These high quality transcriptomic data from N and S plants therefore provide a basis for identifying the key genes involved in tomato dwarfism.

### Differentially expressed genes between normal and stunted tomato pools

**Fig 3:**
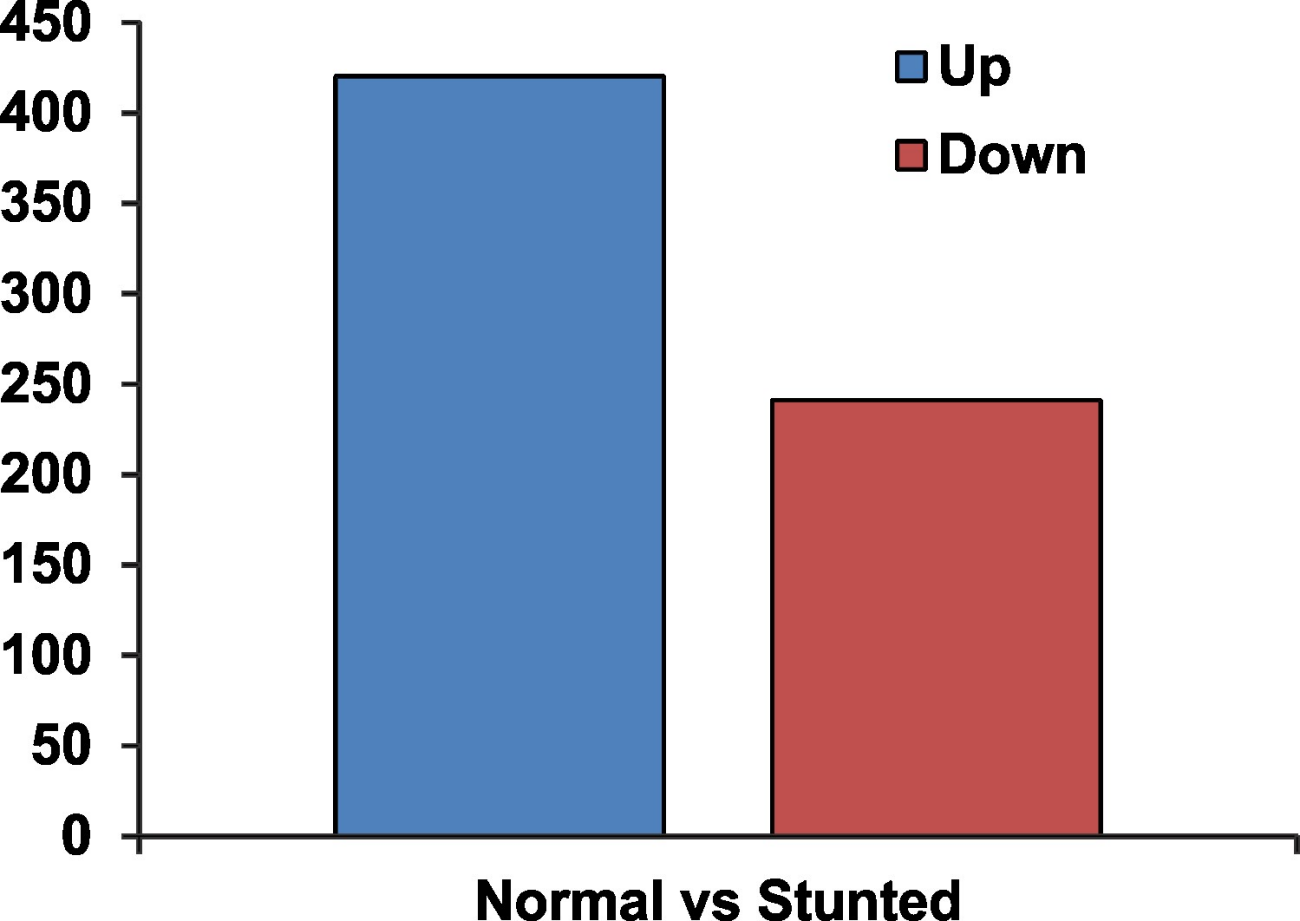
Number of up-regulated and down-regulated genes between normal (N) and stunted (S) cherry tomato cv. ‘Minichal’.

**Fig 4:**
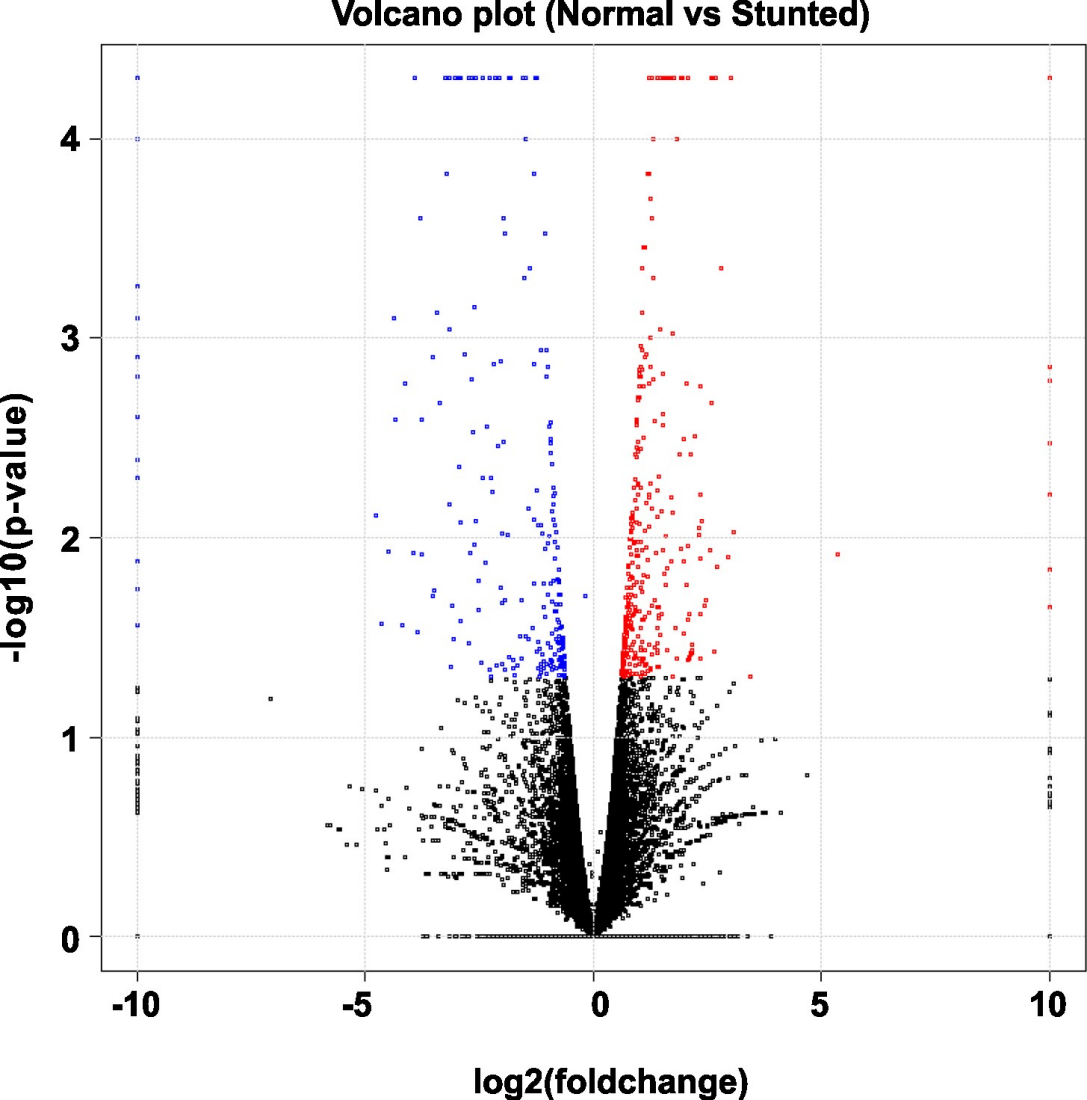
Volcano plot of differentially expressed genes (DEGs) between normal (N) and stunted (S) cherry tomato cv. ‘Minichal’. X-axis and y-axis represent log2 fold-change differences between the compared samples and statistical significance as the negative log of DEG P-values, respectively. The significantly up-regulated and down-regulated genes are indicated with red and blue dots, respectively, while non-significant genes are shown as black dots.

A total of 661 differentially expressed genes (DEGs) between N and S tomato plants were identified using the R package DEGseq (S2 Table) [30]. Of these DEGs, 420 genes were up-regulated, and 241 genes were down-regulated in S versus N (Fig 3). However, 32 DEGs were found to be expressed in S only, and 108 in N only (S2 Table). The distribution of up-regulated and down-regulated genes is shown using a volcano plot (Fig 4).

### Functional classification of DEGs

**Fig 5:**
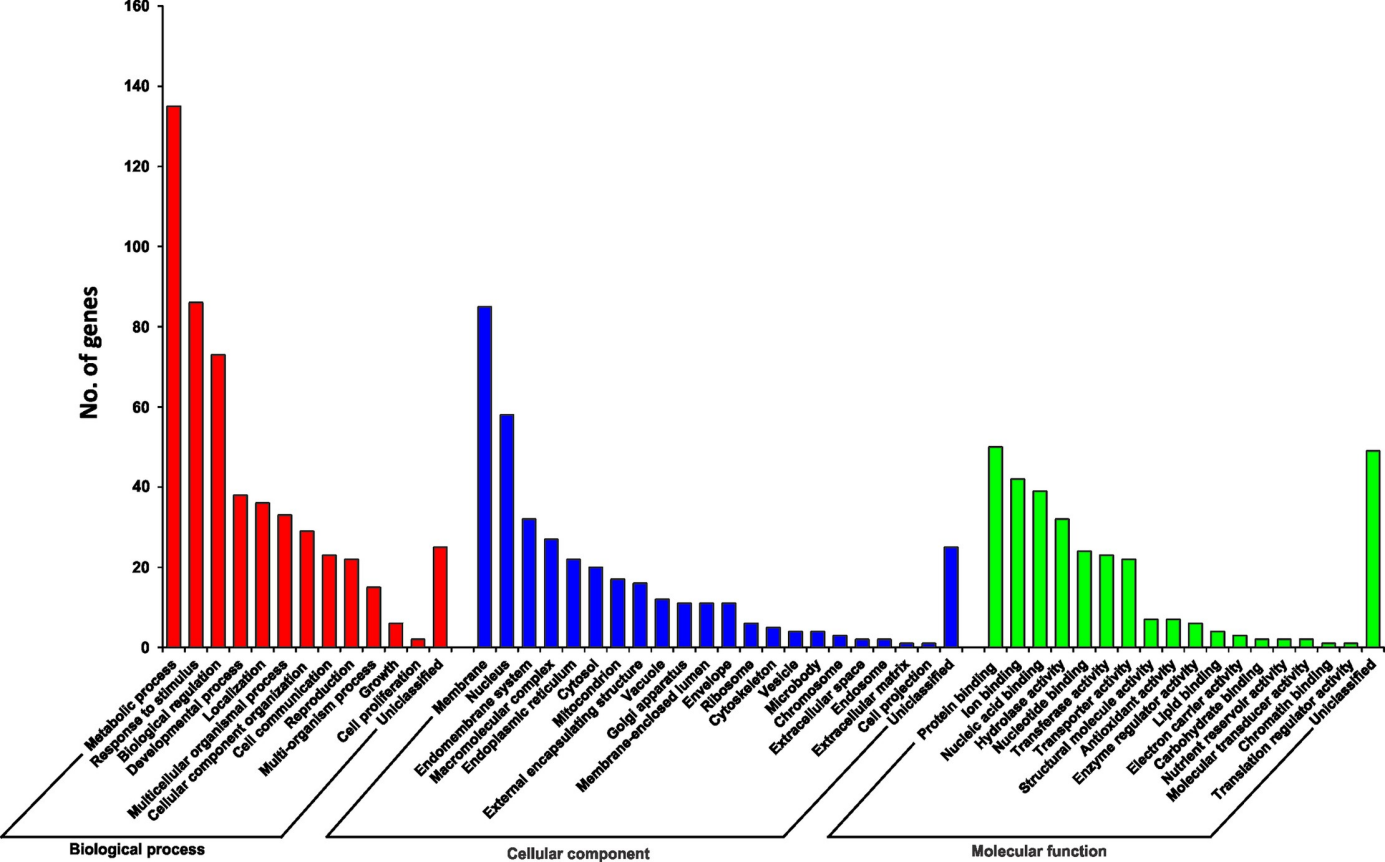
Gene ontology (GO) classification of differentially expressed genes between normal (N) and stunted (S) cherry tomato plants (cv. ‘Minichal’).

Using GO analysis, DEGs were classified into three main categories: biological processes, cellular components, and molecular functions; and 50 functional groups (Fig 5). In the biological process category, ‘metabolic process’ (GO:0008152), ‘response to stimulus’ (GO:0050896), and ‘biological regulation’ (GO:0065007) were the most important GO terms identified; in the cellular components category, ‘membrane’ (GO:0016020) and ‘nucleus’ (GO:0005634) were most important; and in the molecular function category, ‘protein binding’ (GO:0005515), ‘ion binding’ (GO:0043167), ‘nucleic acid binding’ (GO:0003676), and ‘hydrolase activity’ (GO:0016787) were most frequently identified.

**Fig 6:**
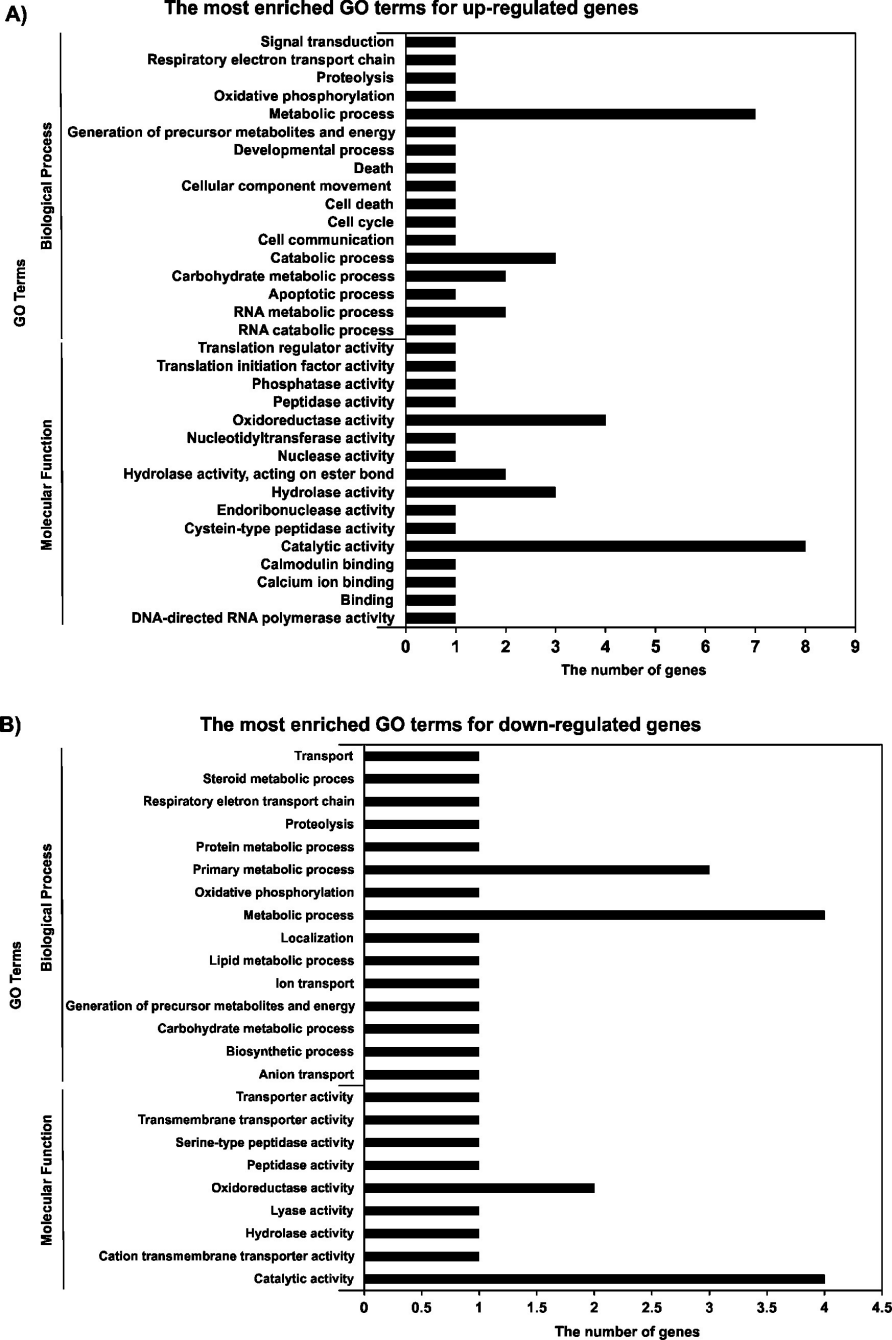
**GO enrichment of up-regulated (A) and down-regulated (B) genes in leaves of stunted (S) and normal (N) cherry tomato plants (cv. ‘Minichal’).** The *P*-value was corrected as *P* < 0.5.

To obtain insight into the biological significance of identified DEGs, GO enrichment analysis was performed using the Gene Ontology database (http://www.geneontology.org/). Enriched GO terms for genes that were up-regulated and down-regulated between N and S tomato plants are shown in Fig 6. GO enrichment analysis revealed that ‘catalytic activity’ and ‘metabolic process’ were the most often enriched GO terms for both up-regulated and down-regulated genes (S3 Table).

Identified DEGs were also subjected to functional annotation clustering at the highest level of stringency using the DAVID database (https://david.ncifcrf.gov/). The analysis showed 22 clusters (S4 Table) with an enriched score ranging from 0.02 to 2.37. Of these, six clusters had an enrichment score greater than 1.0 (Table 2). The most enriched terms were ‘steroid biosynthesis’, ‘WRKY transcription factor’ (TF), ‘DNA damage/repair’, ‘tetratricopeptide repeat’ (TPR), ‘MADS-box TF’, and ‘mitogen-activated protein kinases’ (MAPK).

**Table 2:** Functional annotation clustering of differentially expressed genes (DEGs).

Category	Term	Count	*P*-value	Fold enrichment	Benjamini	FDR
**Annotation cluster 1 (enrichment score: 2.37)**						
UP_KEYWORDS	Sterol metabolism	4	0.002	15.289	0.052	2.723
UP_KEYWORDS	Sterol biosynthesis	4	0.002	15.289	0.052	2.723
UP_KEYWORDS	Steroid biosynthesis	4	0.007	10.084	0.096	8.651
GOTERM_BP_DIRECT	GO:0016126~sterol biosynthetic process	4	0.010	9.024	0.715	12.631
**Annotation cluster 2 (enrichment score: 1.39)**						
SMART	SM00774:WRKY	4	0.029	5.923	0.769	24.581
INTERPRO	IPR003657:DNA-binding WRKY	4	0.034	5.608	0.987	38.225
UP_SEQ_FEATURE	DNA-binding region:WRKY	4	0.069	4.179	0.998	62.629
**Annotation cluster 3 (enrichment score: 1.25)**						
UP_KEYWORDS	DNA repair	5	0.031	4.232	0.188	32.221
UP_KEYWORDS	DNA damage	5	0.037	4.003	0.203	37.264
GOTERM_BP_DIRECT	GO:0006281~DNA repair	5	0.157	2.384	0.963	90.610
**Annotation cluster 4 (enrichment score: 1.18)**						
UP_KEYWORDS	TPR repeat	4	0.0212	6.7709	0.1591	23.4605
INTERPRO	IPR019734:Tetratricopeptide repeat	4	0.1040	3.5289	0.9970	77.9970
INTERPRO	IPR013026:Tetratricopeptide repeat-containing domain	4	0.1269	3.2232	0.9962	84.6116
**Annotation cluster 5 (enrichment score: 1.17)**						
UP_SEQ_FEATURE	domain:MADS-box	4	0.025	6.322	0.988	28.834
SMART	SM00432:MADS	4	0.067	4.223	0.823	48.634
INTERPRO	IPR002100:Transcription factor, MADS-box	4	0.090	3.756	0.997	72.901
GOTERM_BP_DIRECT	GO:0045944~positive regulation of transcription from RNA polymerase II promoter	4	0.147	3.008	0.961	88.971
**Annotation cluster 6 (enrichment score: 1.07)**						
INTERPRO	IPR002487:Transcription factor, K-box	3	0.058	7.675	0.996	55.937
UP_SEQ_FEATURE	domain:K-box	3	0.074	6.604	0.996	65.226
GOTERM_BP_DIRECT	GO:0000165~MAPK cascade	3	0.144	4.476	0.976	88.369

DEG pathway analysis was done using DAVID and the KEGG pathway database, using default threshold parameters except for EASY, which was set at >0.3. The results indicated that ‘steroid biosynthesis’, ‘homologous recombination’, ‘mismatch repair’, ‘DNA replication’, ‘protein export’, ‘glucosinolate biosynthesis’, ‘vitamin B6 metabolism’, ‘nucleotide excision repair’, ‘2-oxocarboxylic acid metabolism’, ‘zeatin biosynthesis’, and ‘cutin, suberin, and wax biosynthesis’ were highly enriched pathways (Table 3), with the most significantly enriched pathways being ‘homologous recombination’ and ‘steroid biosynthesis’.

**Table 3:** KEGG pathway enrichment of differentially expressed genes between N and S.

Sl. no.	Pathway name	No. of genes	*P*-value	Benjamini
1	ath00100:Steroid biosynthesis	3	0.056	0.794
2	ath03440:Homologous recombination	4	0.025	0.748
3	ath03430:Mismatch repair	3	0.071	0.741
4	ath03030:DNA replication	3	0.109	0.795
5	ath03060:Protein export	3	0.116	0.743
6	ath00966:Glucosinolate biosynthesis	2	0.127	0.714
7	ath00750:Vitamin B6 metabolism	2	0.147	0.714
8	ath03420:Nucleotide excision repair	3	0.183	0.750
9	ath01210:2-Oxocarboxylic acid metabolism	3	0.203	0.750
10	ath00908:Zeatin biosynthesis	2	0.203	0.714
11	ath00073:Cutin, suberin and wax biosynthesis	2	0.265	0.785

### Expression pattern of genes related to homologous recombination, steroid and cytokinin/zeatin biosynthesis

**Fig 7:**
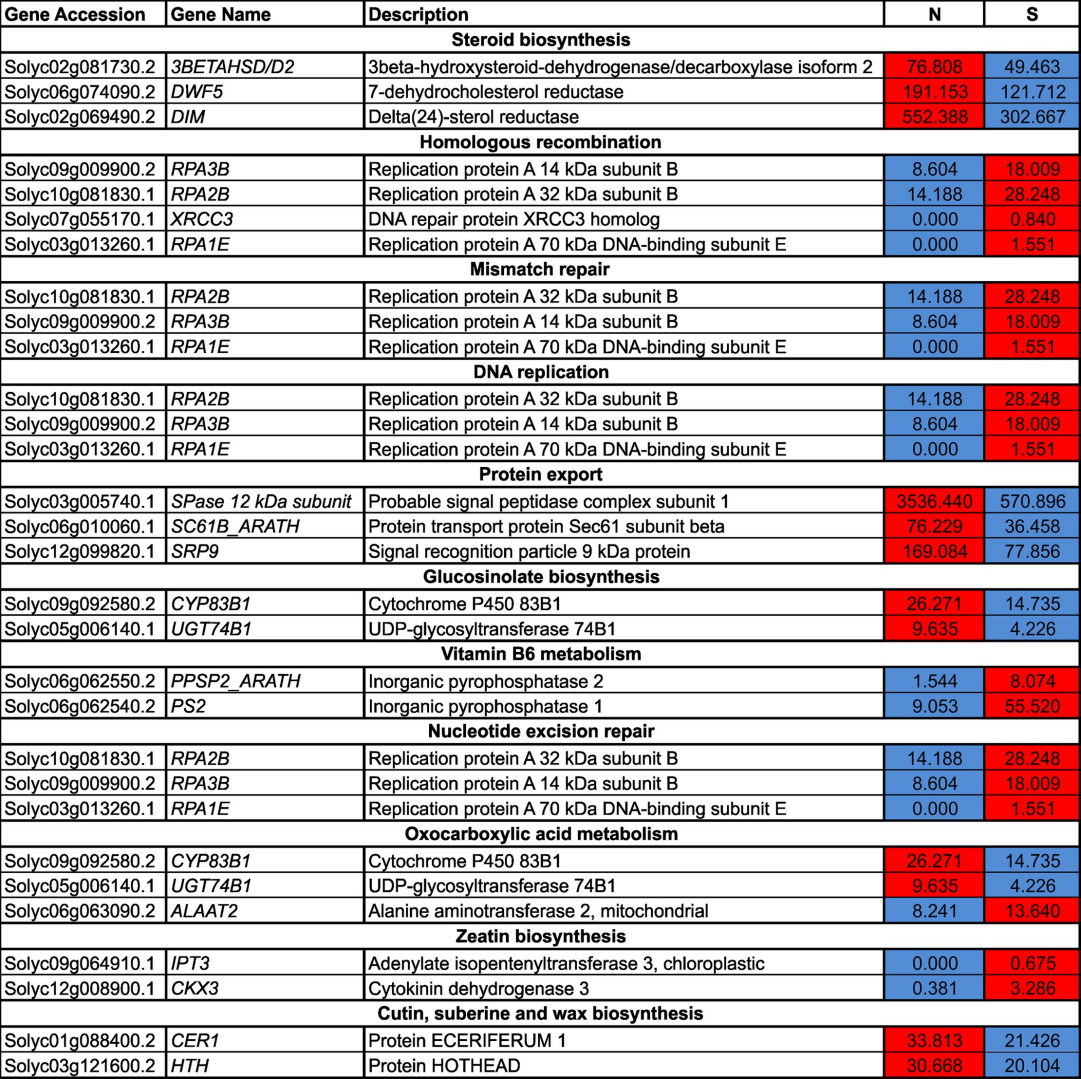
Heatmap illustration of the expression of genes involved in homologous recombination and steroid biosynthesis in normal (N) and stunted (S) cherry tomatoes of the cultivar ‘Minichal’. FPKM values were obtained from RNA-seq data. Red and blue colors represent the maximum and the minimum values, respectively.

Transcript levels of four genes, *RPA3B* (Solyc09g009900.2), *RPA2B* (Solyc10g081830.1), XRCC3 (Solyc07g055170.1) and *RPA1E* (Solyc03g013260.1)—all related to homologous recombination—were up-regulated in stunted (S) ‘Minichal’ cherry tomatoes compared to those with a normal (N) phenotype. On the contrary, three genes related to the steroid biosynthetic pathway, *3BETAHSD/D2* (Solyc02g081730.2), DWF5 (Solyc06g074090.2), and DIM (Solyc02g069490.2), were down-regulated in S compared to N (Fig 7). The gene *adenylate isopentenyl transferase 3* (*IPT3*), which is involved in cytokinin biosynthesis, and *cytokinin oxidase 3* (*CKX3*), which catalyzes the degradation of cytokinins, were up-regulated in S compared to N (Fig 7), although expression of *CKX3* was higher than that of the *IPT3*.

### Expression patterns of other hormone-related genes

**Fig 8:**
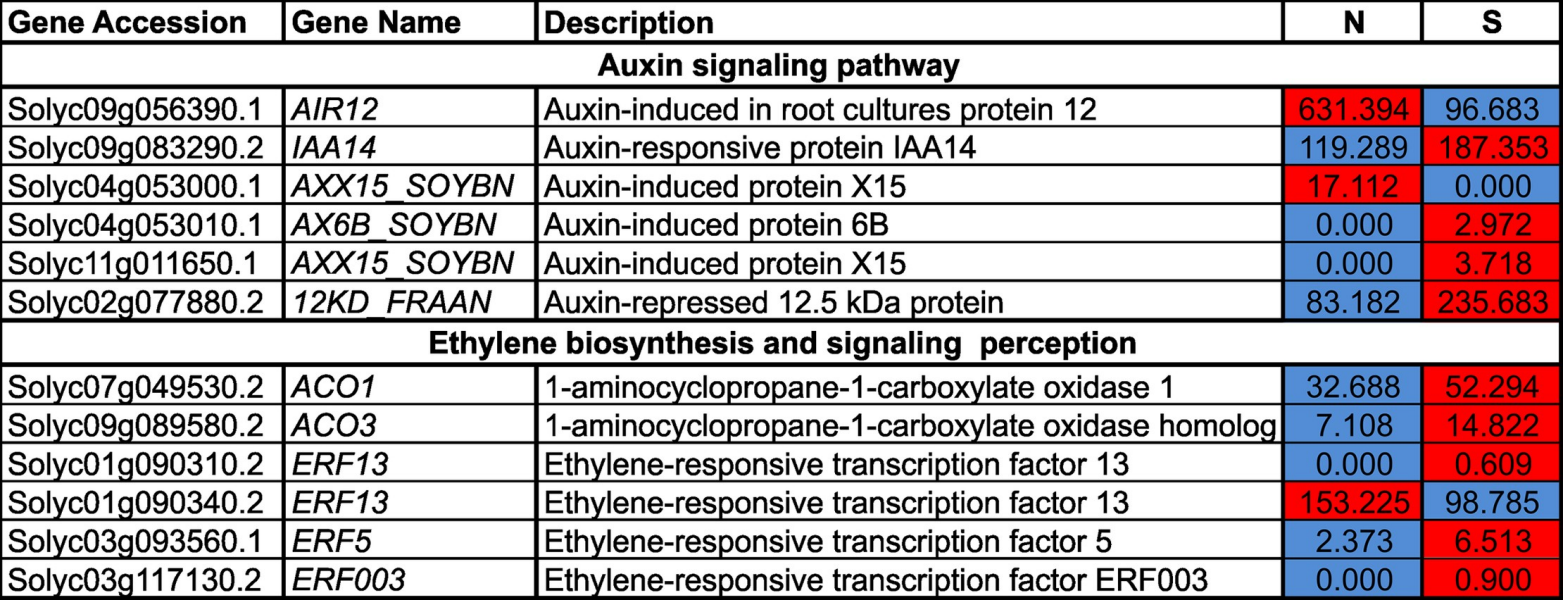
Heatmap illustration of the expression of auxin and ethylene signaling perception genes in normal (N) and stunted (S) cherry tomatoes of the cultivar ‘Minichal’. FPKM values were obtained from RNA-seq data. Red and blue colors represent the maximum and minimum values, respectively.

Among the DEGs, four genes related to the auxin signaling pathway, *IAA14* (Solyc09g083290.2), *AX6B_SOYBN* (Solyc04g053010.1), *AXX15_SOYBN* (Solyc11g011650.1), and *12KD_FRAAN* (Solyc02g077880.2), were up-regulated, and two genes, *AIR12* (Solyc09g056390.1), and *AXX15_SOYBN* (Solyc04g053000.1), were down-regulated in S compared to N (Fig 8). Two ethylene biosynthetic genes, *1-aminocyclopropane-1-carboxylate oxidase 1* (*ACO1*), and *1-aminocyclopropane-1-carboxylate oxidase 3* (*ACO3*), were highly expressed in S compared to N (Fig 8). Furthermore, *ethylene responsive factor* (*ERF*) genes that lie downstream of the ethylene signaling pathway were also differentially expressed. Among these downstream genes, *ERF13* (Solyc01g090340.2) was the most highly expressed and was down-regulated in S compared to N. Expression of the remaining three *ERFs* was very low, and two of these *(ERF003*, Solyc03g117130.2, and *ERF13*, Solyc01g090310.2) were not expressed in ‘N at all.

### Expression patterns of WRKY TF, MADS-box TF, MAPK and TPR-related genes

**Fig 9:**
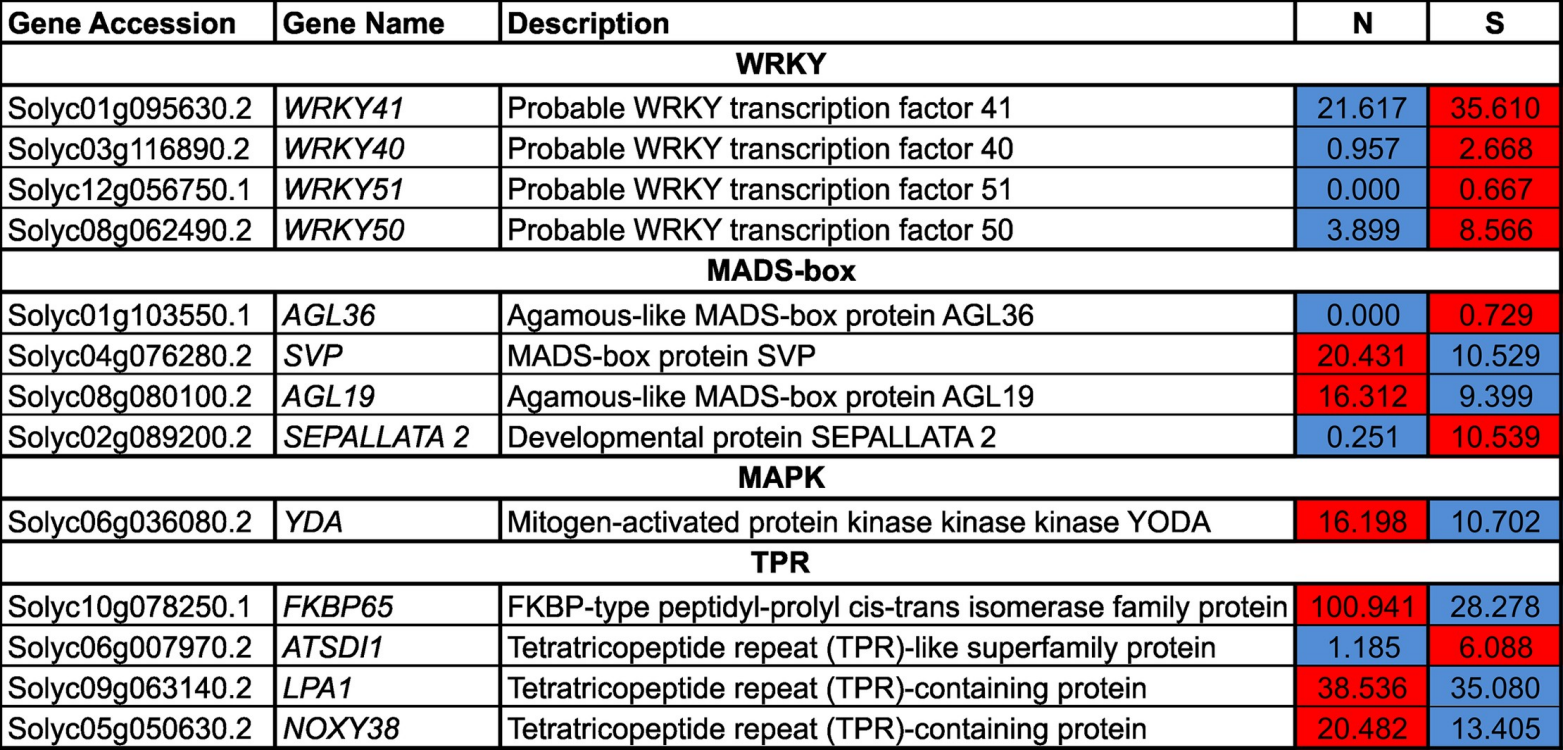
Heatmap illustration of the expression of WRKY, MADS-box, MAPK, and TRP TFs in normal (N) and stunted (S) cherry tomatoes of the cultivar ‘Minichal’. FPKM values were obtained from RNA-seq data. Red and blue colors represent the maximum and minimum values, respectively.

The expression of four WRKY TF genes, *WRKY 40* (Solyc03g116890.2), *WRKY 41* (Solyc01g095630.2), *WRKY 50* (Solyc08g062490.2), and *WRKY 51* (Solyc12g056750.1), were up-regulated in S compared to N (Fig 9). Among the MADS-box TF genes, *AGL36* (Solyc01g103550.1), and *SEPALLATA 2* (Solyc02g089200.2) were up-regulated, while *SVP* (Solyc04g076280.2), and *AGL19* (Solyc08g080100.2), were down-regulated in S compared to N (Fig 9). The *YDA* (Solyc06g036080.2) gene, which encodes a mitogen-activated protein kinase (MAPK), was down-regulated in S (Fig 9). Transcript levels of three *tetratricopeptide repeat (TPR)-like* genes, *FKBP65* (Solyc10g078250.1), *LPA1* (Solyc09g063140.2), and *NOXY38* (Solyc05g050630.2), were down-regulated, while *ATSDI1* (Solyc06g007970.2) was up-regulated in S compared to N (Fig 9).

### Validation of RNA-seq data

**Fig 10:**
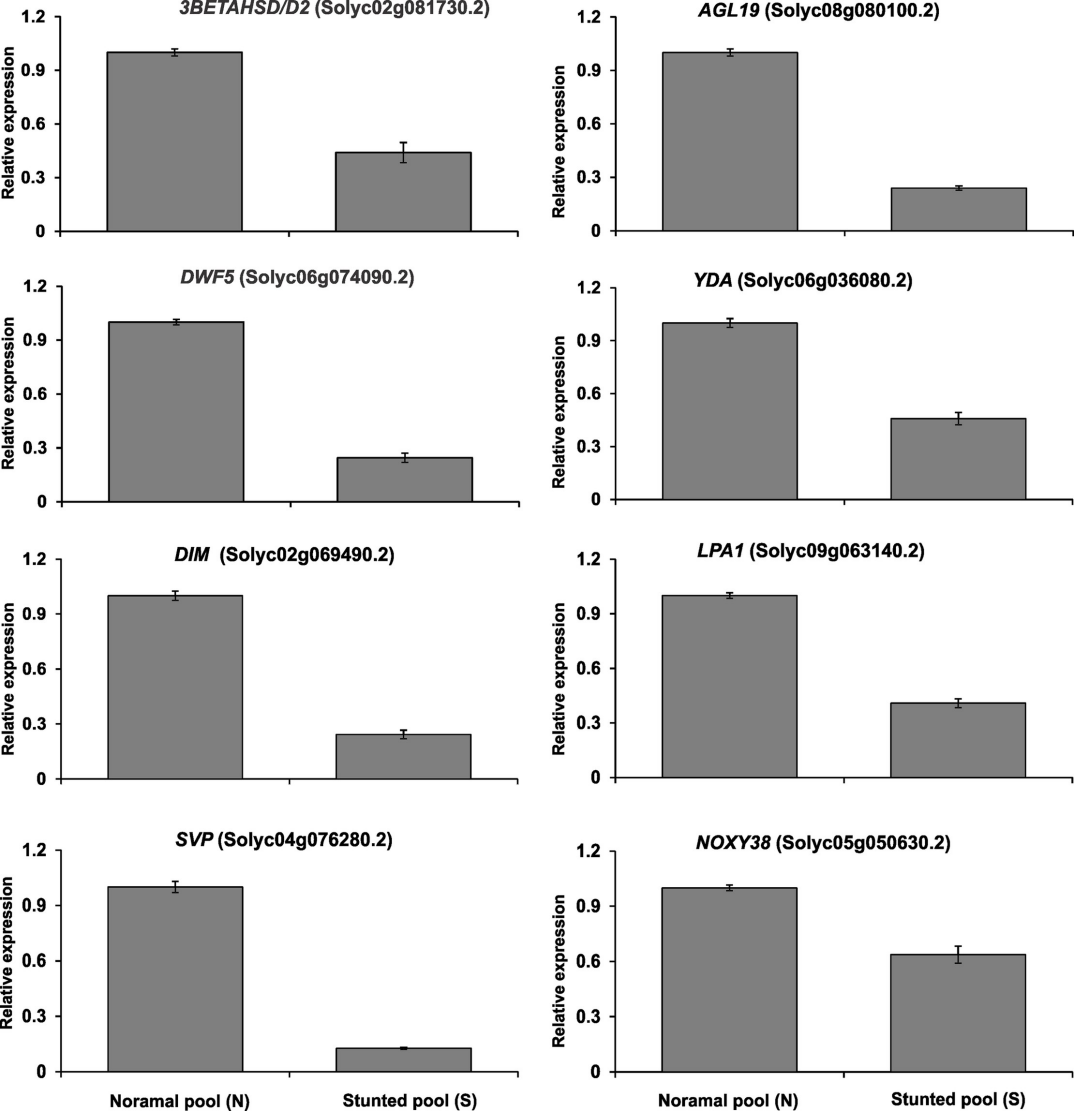
Validation of differentially expressed genes between normal (N) and stunted (S) cherry tomatoes of the cultivar ‘Minichal’ by qRT-PCR. Error bar indicates ±SE of the means of three replicates.

To test the reliability of RNA-seq results, qRT-PCR was used to measure the expression of eight genes with the same RNA samples used for RNA-seq. Relative expression of the tested genes was consistent with the RNA-seq data (Fig 10), confirming the efficiency and accuracy of the RNA-seq experiments.

## Discussion

Deep sequencing-based RNA-seq technology has made it possible to rapidly analyze large genomic datasets and quantify transcriptomes [33]. This high-throughput, next-generation sequencing technology has become a powerful tool for analyzing transcriptomes, and has been successfully used for both human and plant transcriptomes [34]. Global gene expression patterns can be determined using RNA-seq in samples of tissues at different developmental stages, with contrasting characteristics, or in response to different environmental stimuli [33,35,36]. In this study, we observed a stunted phenotype of the cherry tomato cv. ‘Minichal’, which is characterized by alterations in plant height, leaf size/shape, and fruit size/shape compared to the normal phenotype. We used RNA-seq to profile the transcriptomes of normal (N) and stunted (S) cherry tomatoes of this cultivar. We obtained almost 115.450 million high-quality clean reads, which were assembled into 35,216 transcripts (Table 1).

Our results identified 661 DEGs between the pooled RNA of N and S tomato plants (S2 Table). Subsequently, GO enrichment revealed that ‘metabolic process’ and ‘catalytic activity’ were the most enriched GO terms for both up-regulated and down-regulated genes between N and S (S3 Table).

To obtain further insight into the biological functions of these DEGs, GO functional annotation and KEGG pathway enrichment analysis were performed using the DAVID tool. Functional annotation clustering of DEGs revealed that the most enriched GO terms were associated with the sterol biosynthesis process (GO:0016126; enrichment score 2.37) (Table 2). KEGG pathway enrichment also indicated that ‘steroid biosynthesis’ and ‘homologous recombination’ were the most enriched pathways (Table 3). These results clearly suggest that genes related to steroid biosynthesis might be involved in dwarfism in S tomatoes.

Several studies have been conducted on plant dwarfism. Dwarfism is sometimes advantageous; for example in cereal crops—specifically rice and wheat, where lodging decreases crop productivity [37]. However, in tomato, dwarfism is deleterious because it reduces both quality and productivity. Plant dwarfism results from many genetic defects, mostly associated with hormone biosynthesis and perception [12]. Functional analysis of several genes related to dwarfism has previously been reported, including genes related to BR and GA biosynthesis and perception in different plant species [22,28,38–41].

BRs play significant roles in plant growth and development, and are biosynthesized via multiple parallel pathways starting with the precursor campesterol [15,24,42,43]. Defects in the BR biosynthesis/signaling cause dwarfism in plants [13]. For example, in *Arabidopsis*, *dwarf5* (*dwf5*) mutants have a mutation in the gene for the enzyme 7-dehydrocholesterol reductase, which disrupts the sterol Δ^7^ reduction step and leads to dwarfism [25]. Likewise in *Arabidopsis*, dwarfism occurs in *dwf4* mutants, which have a mutation in the gene encoding steroid 22α hydroxylase (CYP90B1), which is involved in 22α‐hydroxylation of the BR pathway [24].

**Fig 11:**
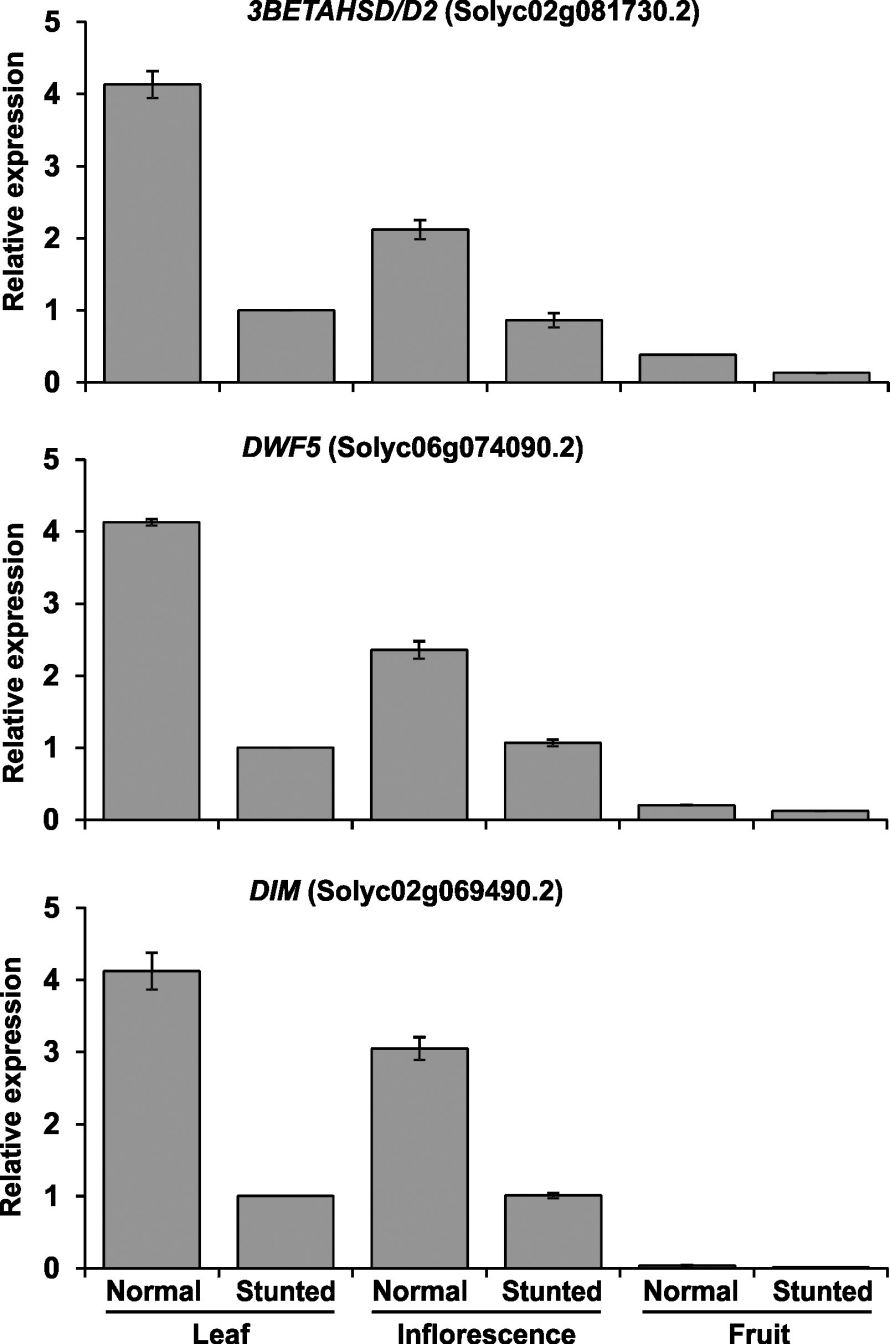
Relative expression of three genes related to steroid biosynthesis in leaf, inflorescence, and fruit of normal (N) and stunted (S) plants of the cherry tomato cv. ‘Minichal’. Error bar indicates ±SE of the means of three replicates.

In this study, three DEGs, *3beta-hydroxysteroid-dehydrogenase* (*3BETAHSD/D2*, Solyc02g081730.2), *7-dehydrocholesterol reductase* (*DWF5*, Solyc06g074090.2), and *delta(24)-sterol reductase* (*DIM*, Solyc02g069490.2)—all related to the steroid hormone biosynthesis pathway—were down-regulated in S compared to N plants (Figs 7 and 10). We also checked the expression patterns of these three genes in leaf, inflorescence, and fruit tissues of the cherry tomato cv. ‘Minichal’ (Fig 11). The result indicated that the expression of these steroid biosynthesis genes was higher in N than S plants. In N, expression was highest in leaf and lowest in fruit, while in S, expression was similar in leaves and inflorescences, but was drastically reduced in fruits.

**Fig 12:**
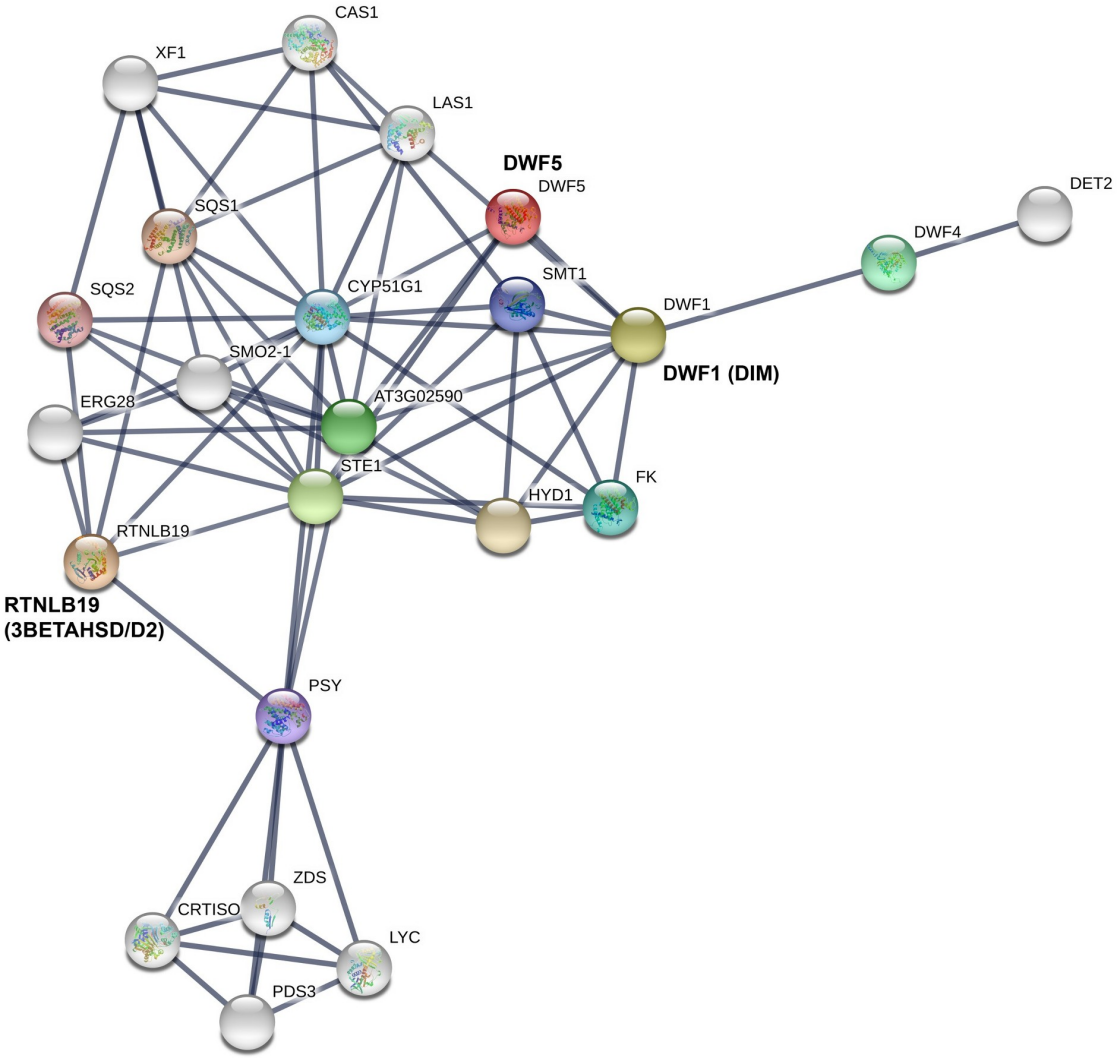
Protein network interaction of differentially expressed steroid pathway-related genes and proteins analyzed using STRING (http://string.embl.de).

The protein interaction network of three steroid biosynthesis-related genes, *3beta*-*hydroxysteroid-dehydrogenase* (*3BETAHSD/D2*), *7-dehydrocholesterol reductase* (*DWF5*), and *delta(24)-sterol reductase* (*DIM*), highlighted their possible contribution to dwarfism of tomato plants (Fig 12). In S cherry tomatoes, these genes might be involved in dwarfism by directly or indirectly affecting steroid biosynthesis. In apple plants (*Malus* × *domestica*), colchicine-induced autotetraploid plants showed dwarfism, with decreased levels of indole-3-acetic acid (IAA) and BR compared to diploid plants. Furthermore, digital gene expression analysis of these apple plants revealed that DEGs between them were mostly related to IAA and BR biosynthesis pathways [44]. In *Arabidopsis*, a biosynthetic defect in *dwf1*, which encodes *delta(24)-sterol reductase*, resulted in dwarfism with reduced levels of BR synthesis compared to the wild type [45]. A similar *dwf1* dwarf mutant has been reported in pea [46]. The *dwf5* mutant, which is defective in BR biosynthesis, also showed a dwarf phenotype in *Arabidopsis* [25]. In rice, the dwarf mutant *ebisu dwarf* (*d2*) is deficient in BR biosynthesis and caused dwarfism, but exogenous application of BL restored the normal phenotype [39]. In tomato, the BR biosynthesis-defective mutant *Dwarf* (*D*), which harbors a mutation in *cytochrome P450* (*P450*), exhibits dwarfism, while complementation *35S*::*D* lines restore the normal phenotype [12,40]. Similar dwarf mutant *dumpy* (*dpy*) resulted from the mutation of mutation in the *C-23 steroid hydroxylase* (*cpd*) gene has also been reported in tomato by Kaka et al. [47]. However, our reported genes (*3BETAHSD/D2*, *DWF5* and *DIM*) for dwarfism of cherry tomato are different from those previously reported mutants like *D* and *dpy*. Up-regulation of *cytokinin dehydrogenase 3* (*CKX3*) in S tomatoes led to a higher rate of cytokinin degradation in these plants. Reid et al. [48] reported that the cytokinin content was negatively regulated by the activity of *CKX3* in the root of *Lotus japonicus ckx3* mutants.

The auxin signaling genes *AIR12 (Solyc09g056390*.*1)*, and *AXX15* (*Solyc04g053000*.*1*), but not *IAA14*, were down-regulated in N (Fig 8). This suggests that auxin signaling genes might be affected, leading to defective plant development. A similar result has been reported in tetraploid apple [44].

Previous studies have revealed that GA has an effect on plant growth and development. For example, exogenous treatment of dwarf pea and dwarf maize seedlings with GA3 enhanced longitudinal growth rates [49]. However, we found no DEGs related to GA biosynthesis in this study.

The up-regulation of two *1-aminocyclopropane-1-carboxylic acid oxidase* genes, *ACO1* and *ACO3*, which are involved in the final step of ethylene biosynthesis, suggests higher levels of ethylene production in S tomatoes, which might affect plant growth and development. Ethylene overproduction has been shown to inhibit plant growth in *Arabidopsis* [50,51].

The ‘short vegetative phase’ (SVP) group of MADS-box genes, such as *OsMADS22*, *OsMADS47*, and *OsMADS55*, have been shown to act as negative regulators of BR responses in rice [52]. The double and triple RNAi plants (*OsMADS22*–*OsMADS55* and *OsMADS22*–*OsMADS47*–*OsMADS55*, respectively) showed reduced stem elongation. Unexpectedly, in this study, we also found that the expression of the MADS-box genes *SVP* (*Solyc04g076280*.*2*) and *AGL19* (*Solyc08g080100*.*2*) was down-regulated, and *AGL36* (*Solyc01g103550*.*1*) and *SEPALLATA2* (*Solyc02g089200*.*2*) were up-regulated in S compared to N plants (Fig 9). Overexpression of *OsMADS1* causes dwarfism in rice via irregular activation of BR and GA synthesis pathways [53].

Kim et al. [54] demonstrated that BR controls stomatal development by activating *mitogen-activated protein kinase kinase kinase* (*MAPKKK*) in *Arabidopsis*. Likewise, we found that *MAPKK* was up-regulated in S compared to N tomatoes (Fig 9).

## Conclusions

We conducted comparative transcriptome analysis using normal and stunted plants of the cherry tomato cv. ‘Minichal’. DEGs related to steroid biosynthesis may be involved in dwarfism in this tomato cultivar. To best of our knowledge, this is the first comparative transcriptome analysis for plant dwarfism in tomato. Our results provide insight into the molecular mechanism of dwarfism and lay the foundation for future studies in related species.

## Supporting information

S1 Table Primers used for qRT-PCR validation. (XLSX)

S2 Table Differentially expressed genes between normal (N) and stunted (S) tomato plants. (XLSX)

S3 Table GO terms overrepresented in up-regulated and down-regulated genes, and number of genes belonging to each term. (XLSX)

S4 Table Twenty-two functional annotation clusters of DEGs. (XLSX)
