# Correction: Comparative transcriptome analysis provides insights into dwarfism in cherry tomato (*Solanum lycopersicum* var. *cerasiforme*)

**DOI:** 10.1371/journal.pone.0211396

**Published:** 2019-01-23

**Authors:** The PLOS ONE Staff

In the Funding section, the grant number from the funder The Golden Seed Project is listed incorrectly. The correct grant number is: 213007-05-3-CG100. The publisher apologizes for this error.
